# Reamed intramedullary exchange nailing: treatment of choice of aseptic femoral shaft nonunion

**DOI:** 10.1186/s13018-014-0088-1

**Published:** 2014-10-10

**Authors:** Christian Hierholzer, Claudio Glowalla, Michael Herrler, Christian von Rüden, Sven Hungerer, Volker Bühren, Jan Friederichs

**Affiliations:** Trauma Center Murnau, Berufsgenossenschaftliche Unfallklinik Murnau, Prof.-Kuentscher-Str. 8, 82418 Murnau, Germany

**Keywords:** Femoral nonunion, Exchange nailing, Reamed intramedullary nailing

## Abstract

**Background:**

The aim of this study was to evaluate a standardized method of treatment of femoral nonunion of the isthmal femur excluding non-united metaphyseal fractures.

**Methods:**

Between 2003 and 2010, 72 consecutive patients with nonunion of the femoral shaft were operated using a standardized protocol in our trauma department and followed up for successful union and functional result.

**Results:**

Osseous healing was observed in 71 patients (98%). Only one patient was lacking bone healing following a time period of 24 months after the first exchange nailing and 5 months after the second exchange nailing. In 59 patients (82%), uneventful and timely bone healing after exchange nailing was detected. In 18% of patients (*n* = 13), delayed bone healing was observed and required additional therapy. In the majority of patients (61%), bone healing occurred within the first 2 to 5 months, only 18% of patients’ duration of bone healing exceeded 8 months. In 62 patients (86%), no relevant or clinically apparent leg-length discrepancy prior to and after exchange nailing was detected as well as no significant axis deviation or malrotation. Functional studies including simple clinical gait and standing analysis, return to activities of daily life, return to sports activities, and return to work were all reached on a satisfying level.

**Discussion:**

Reamed intramedullary exchange nailing as described in this study is the treatment of choice for aseptic femoral shaft nonunion with a high rate of bone healing and a low rate of complications including length discrepancy or malrotation and a good functional outcome.

## Background

For fractures of the femoral shaft, operative treatment is generally recommended, and intramedullary nailing is considered the treatment of choice [1-4]. Many risk factors for nonunion have been identified such as the use of small diameter, unreamed nails [2], nonsteroidal anti-inflammatory drugs [5], open fractures, and smoking [1,6,7]. Although union rates are reported to be as high as 85%–100% [1-4], nonunion does occur mostly in the form of aseptic hypertrophic nonunion. The management of these non-united femoral shaft fractures or delayed union still represents a challenge for the treating surgeon.

According to the literature, exchange nailing remains the treatment of choice for aseptic, non-comminuted nonunion of the femoral diaphysis following primary intramedullary nailing [8-11]. The procedure involves the removal of the previously inserted nail, the over reaming of the shaft with an enlargement of 1–3 mm, and the replacement of the nail with an increased diameter. Open application of autologous bone grafting usually is not required, but internal bone grafting by the reaming material is successfully utilized for stimulation of osteogenesis [12,13].

The initially reported success rates of exchange reamed intramedullary nailing of femoral nonunion were as high as 100% [8-12,14]. However, two studies questioned the effectiveness of this technique demonstrating failure rates of 27% and 42%, respectively [15,16]. Several reasons for these diverging results have been suggested including technical improvements in implant design, and the introduction of interlocking screws leading to intramedullary treatment of complex fractures and thus, to more tenacious, complex nonunion [11,14].

The aim of this study was to evaluate a standardized method of treatment of isthmal femoral nonunion excluding non-united non-isthmal, metaphyseal fractures. Between 2003 and 2010, 72 patients with aseptic nonunion of the femoral shaft were operated using a standardized protocol in our trauma department and followed up for successful union and functional result.

## Methods

Between 2003 and 2010, 72 consecutive patients with aseptic femoral shaft nonunion were treated at the Trauma Center Murnau, a level I trauma center in Germany. A prospective case series was performed, and all patients were included in this study who had been treated with intramedullary nailing of a femoral shaft fracture and had developed femoral shaft nonunion. The study was conducted in compliance with the Helsinki Declaration and according to the guidelines and the approval of the Ethics Committee of the Bavarian Medical Board. Nonunion was defined clinically and radiologically according to the literature [17,18] after 6 or even 9 months of missing union in fracture treatment without progression towards healing over 3 consecutive months. However, in some cases (20%), treatment decisions were not based on a specific number of months but on clear loss of progress of bone healing so we did not wait for 6 or even 9 months to diagnose fracture nonunion. Clinical signs included persistent pain with weight bearing, and radiologically, nonunion formation was determined as lack of radiographic bridging of at least three out of four cortices assessed on a.p. and lateral conventional radiologic views. In cases of doubt, a CT scan was performed to detect radiological nonunion.

Of the 72 patients included in this study, 48 were referred for treatment of the femoral shaft nonunion from outside institutions and 28 were initially treated at our institution. The study group consisted of 56 male and 16 female patients with a median age of 46 years (range 18–69 years). Forty-nine patients (68%) suffered femur fracture as part of polytrauma injury (ISS >16) and 23 patients (32%) as a monotrauma (Table 1).

**Table 1:** **Summary of clinical characteristics of 72 patients with an aseptic nonunion of the femoral shaft**

**Parameters**	**Values**
Age (year), mean (range)	46 (18–69)
Male/female	56/16 (78%/22%)
Initial polytrauma/monotrauma	49/23 (68%/32%)
AO-classification	
Type A	50 (70%)
Type B	11 (15%)
Type C	11 (15%)
Closed fractures	50 (70%)
Open fractures	
I°	11 (15%)
II°	11 (15%)
III°	0
Index operation	
Non-reamed	47 (65%)
Reamed	25 (35%)
Median time to revision surgery	11 months

Applying AO-classification criteria, 50 patients (70%) had type A fractures, 11 patients (15%) type B fractures, and 11 patients (15%) type C fractures. Fractures of the proximal and distal non-isthmal region of the femoral bone were excluded from the study; the majority of initial fractures (54%) were observed in the median isthmus, 13 patients (18%) presented with fractures of the proximal part of the isthmal region, and 20 patients (28%) in the distal part. Fifty fractures (70%) were classified as closed fractures, 11 (15%) were first degree open fractures according to the Gustilo-Anderson classification, 11 (15%) second degree, and no third degree initial fractures were observed. In the index operation in 47 cases (65%), non-reamed nails were utilized and 25 patients were treated with reamed nails.

Nonunion was classified as hypertrophic in 90% (65 patients) and atrophic/oligotrophic in 10% (7 patients). Median time between index operation and revision surgery was 11 months, for 33 patients (46%) more than 12 months, for 24 patients (34%) between 6 and 12 months and less than 6 months for 15 patients (20%).

### Surgical technique

The patient was positioned in a lateral position on a radiolucent operating table. Bacterial swaps from the extracted nail and reaming material were obtained for several bacterial cultures. All patients with positive cultures were excluded from the study.

After removal of the nail, a guide wire was gently bent at the tip and was inserted into the intramedullary canal. Care was taken to precisely position the guide wire in the center of the femoral intercondylar region assessed by a.p. and lateral radiologic views; thus, a physiological axis alignment of the femoral shaft can be expected after successful femoral nailing. Sequential reaming was performed with the goal of inserting an exchange nail with an increased diameter of 2 mm compared to the initial nail diameter size. Therefore, in the isthmal region, the intramedullary canal was over reamed with 2 mm more than the determined, final nail diameter. The median nail diameter of the extracted nail was 10 mm (range 9–13 mm) and the inserted exchange nail 12 mm (range 10–17 mm). It was ensured that all exchange nails demonstrated good cortical contact and a snug fit, and that any fracture gap or dehiscence was avoided.

For exchange nailing, T2-femoral-nails (Stryker, Duisburg, Germany) were utilized which offer the possibility of interfragmentary compression. Initially, distal interlocking screws were inserted. For the assessment of femoral torsion, the femoral condyles were imaged in a lateral view with precise projection of both condyles. The C-arm was fixed and then moved in strictly parallel direction until centered over the region of the femoral head. Torsion of the femur was considered acceptable, if the projection of the femoral head was anterior to the axis of the femoral shaft with two thirds of its circumference.

Consecutively, patients received physiotherapy. Weight bearing as tolerated was permitted. A radiologic follow-up study was performed 3 to 7 days following exchange nailing.

### Follow-up

After discharge, patients were followed up at regular office visits every 6 weeks. Clinical assessment of wound healing, condition of soft tissues, and pain with weight bearing was performed; sequential radiologic follow-up studies were requested at regular intervals at 6 and 12 weeks as well as 6 months postoperatively. The median follow-up of patients was 14 months with a range from 12 to 38 months. All radiographs were assessed by a fellow or attending of the Department of Radiology blinded to the patients’ outcome.

### Statistical analysis

Noncontinuous variables were tested using the Fisher exact test. A *p* value of <0.05 was considered statically significant. Statistical analysis was performed using Statistical Package for the Social Sciences (SPSS) 19.0 (SPSS, Chicago, USA).

## Results

In the majority of patients (*n* = 65), the nonunion was classified as hypertrophic, and in the majority of patients, the nonunion site was localized in the mid-shaft area. The typical shape of hypertrophic nonunion offers enough bone contact with a broad interfragmentary surface and bone spikes that get impacted and dentated with application of axial compression. Therefore, the dynamic proximal locking option was utilized in 61 (84%) of patients, and the proximal locking screw was inserted in the slotted proximal dynamic hole accordingly. In seven patients (10%), static locking was used proximally, and in four patients (6%), the advanced locking option was applied with axial compression exerted on the dynamic interlocking bolt followed by insertion of additional locking screw into the static locking hole. Exchange nailing was carried out as a closed procedure without opening of the nonunion site in 63 (88%) of patients, and only in 12% of patients (*n* = 9), an open approach to the nonunion site including resection of fibrous nonunion tissue and correction of axis deviation was performed. The diameter of the extracted nail used for fracture stabilization was measured and demonstrated a median diameter of 11 mm with a range of 9 to 13 mm. The inserted exchange nail used for stabilization of the nonunion or delayed union had a median diameter of 12 mm with a range of 10 to 17 mm.

**Figure 1:**
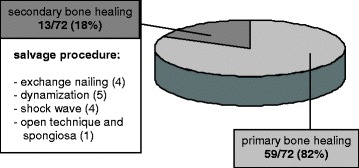
**Distribution of patients with primary and secondary bone healing.** Distribution of patients with primary bone healing after a single operation and secondary bone healing requiring additional therapies following reamed intramedullary exchange nailing of femoral shaft nonunion.

**Figure 2:**
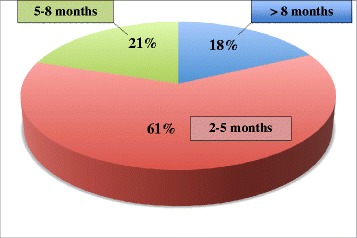
**Time to bone healing of 72 patients with a femoral nonunion treated with reamed intramedullary exchange nailing.**

Osseous healing of the femoral shaft delayed or nonunion was observed in 98% of patients (*n* = 71). Only one patient was lacking bone healing following a time period of 24 months after the first exchange nailing and 5 months after the second exchange nailing. In 59 patients (82%), uneventful bone healing after exchange nailing was detected within an adequate period of time. In 18% of patients (*n* = 13), delayed bone healing occurred requiring additional therapy. Forms of additional therapy are summarized in Figure 1. The time to bone healing of the nonunion was assessed by performing serial x-ray follow-up studies in intervals of 6 weeks. In the majority of patients (61%), bone healing occurred within the first 2 to 5 months, in 21% of patients within 5 to 8 months, and in 18% of patients’ duration of bone healing exceeded 8 months (Figure 2).

Following exchange nailing postoperative complications required therapy in 11 patients. Impaired wound healing at the insertion site of the nail was observed in three patients treated successfully nonsurgically with i.v. antibiotics; in four patients, hematoma formation at the insertion site of the nail occurred treated surgically with local revision and irrigation of soft tissues; and in two patients, unilateral swelling of the lower extremity was observed and deep venous thrombosis was diagnosed. In two patients, breakage of the distal interlocking bolt was observed.

**Figure 3:**
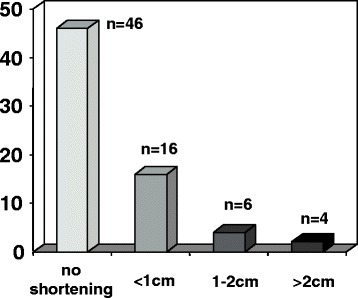
**Leg-length discrepancy after exchange nailing of 72 patients with a femoral nonunion treated with reamed intramedullary exchange nailing.** Note that a variation of 1 cm was accepted as normal.

**Figure 4:**
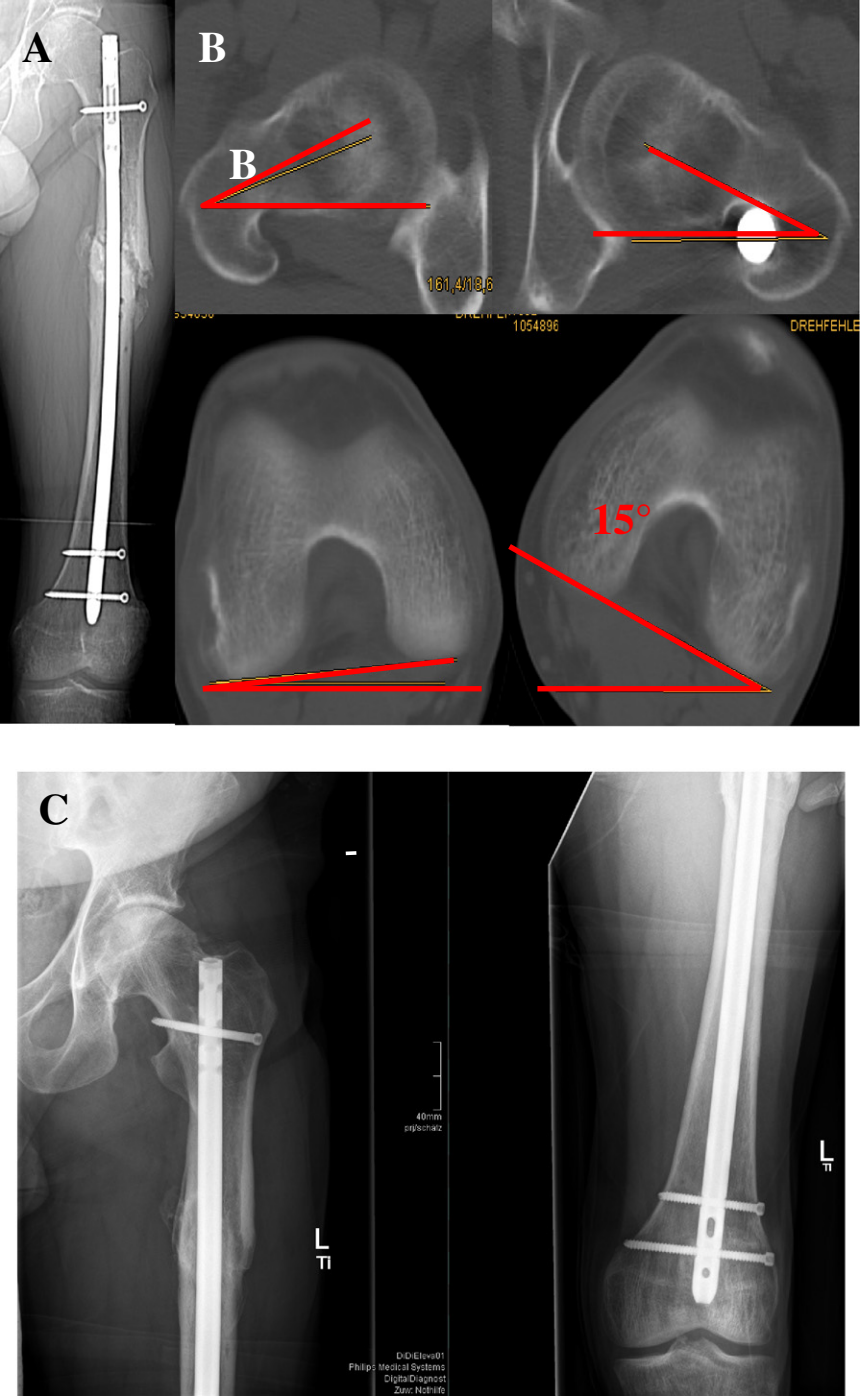
**Clinical example of a patient with a combined varus axis deviation (A) and a rotational difference of 15° (B) prior to reamed intramedullary exchange nailing of a femoral nonunion.** After 6 months, osseous healing could be achieved **(C)**.

Before and after exchange nailing, the leg-length was compared clinically by measuring the distance between the anterior superior iliac spine and the tip of the distal fibula with the patient lying in supine position, as well as by determining the alignment of the pelvic ring with the patient standing in front of the observer and looking for a pelvic tilt. In 62 patients (86%), no relevant or clinically apparent leg-length discrepancy prior to and after exchange nailing was detected. Including an accepted variation of 1 cm, ten patients (14%) presented with a significant difference of leg-length before and after exchange nailing. Results are summarized in Figure 3. Axis alignment was assessed by measuring the mechanical axis of the femur on the digitalized conventional x-ray imaging studies and using standard software. Analysis of the axis deviation in the coronal plane demonstrated varus axis deviation in 7 patients with a median varus of 8° (range 5°–11°), and a valgus axis deviation in 6 patients with a mean of 11° (range 5°–13°). For assessment of the torsional axis deviation, clinical examination of the patient range of motion for internal and external rotation of the hip joint was determined, and in patients who demonstrated a clinical apparent discrepancy in range of motion for external or internal rotation compared to the unaffected hip joint, CT analysis of the femur axis in the axial plane using and comparing predefined landmarks in the trochanteric and femoral neck as well as the condyle regions. In 13 patients, CT scan analysis was offered for exact assessment of torsional alignment, and in 11 patients, the radiological study was performed demonstrating a median external rotation deviation of 17° in 5 patients and a median of internal rotation of 11° in 6 patients. The torsional measurement following exchange nailing demonstrated amelioration in all 11 patients with a median correction of the torsional axis deviation of 10°. A clinical example is shown in Figure 4.

In addition to assessment of bone healing and axis alignment, functional studies including simple clinical gait and standing analysis, return to activities of daily life, return to sports activities, and return to work if applicable were assessed. Subjective contentment and pain assessment by the patient were inquired using a simple questionnaire. The gait was clinically evaluated for any limping pattern, and the patient was requested to perform single leg standing positions, toe and heel standing, as well as the squatting position. Ninety-five percent of the patients demonstrated normal gait pattern with normal rolling function and sequence of steps and without any limping and were able to perform securely and independently various static standing positions. The functional outcome is shown in Table 2.

**Table 2:** **Functional outcome of 72 patients after reamed intramedullary exchange nailing as a treatment of aseptic femoral shaft nonunion**

**Parameters**	**Values**
Normal gait and standing pattern	68/72 (94%)
Sport activities, unrestrained or minimal handicap	63/72 (88%)
No pain or minimal pain	68/72 (94%)
Return to work	61/72 (85%)
Subjective contentment	65/72 (91%)

Outcome was assessed by short questionnaires and clinical gait and standing analyses.

## Discussion

In our study of femoral shaft nonunion, reamed exchange nailing was successfully applied for the treatment of 72 patients and resulted in healing of delayed or nonunion in 98% of the patients. In 82%, closed nonunion treatment was performed without surgical opening of the nonunion site. Reamed exchange nailing and dynamic compression of the nonunion site resulted in increased stability with the possibility of early and unrestricted weight bearing, high patient comfort with little pain and discomfort, as well as good functional outcome. Key steps for successful healing include closed nonunion treatment, correction of axis deviation, limited reaming and biological augmentation by internal reaming graft, and increased rotational and axial stability by insertion of an increased nail diameter and by dynamic compression of the nonunion site. These key steps create a beneficial biological and mechanical environment for bone healing.

The gold standard of treatment of femoral shaft fractures is anterograde intramedullary nailing [1-4] although the rate of nonunion is reported to be between 6.3% and 12.5% [3,9]. The typical nonunion in our cohort was a hypertrophic nonunion with 83% of cases. The characteristics of hypertrophic nonunion include preserved vascularity and intact osteogenesis as indicated by the formation of abundant callus [7]. The predominant problem is the lack of sufficient stability both axially and rotationally. The axial instability may be caused by the application of a small-diameter nail, typically using unreamed technique, which does not provide a snug intramedullary fit. Axial instability is also caused by leaving a fracture gap between the proximal and distal fracture segment without interfragmentary bone contact. Rotational instability may originate from omitting distal interlocking or by application of an insufficient number of distal interlocking screws specifically in cases with a short distal shaft fragment. As a surgical principle, we recommend to secure the short fracture fragment by three interlocking screws. Axis deviation may also contribute to instability. Specifically, varus axis deviation has been shown to be detrimental for fracture healing and should be avoided. Axis deviation may be caused by imprecise surgical technique including incorrect proximal insertion point. Incorrect end point may also result in valgus or varus axis deviation, and therefore, it is critical to place the guide wire perfectly central in the femur condyle region. This is one of the major advantages of reamed nailing compared to unreamed nailing.

As described above, we favor closed nonunion treatment without opening or resection of the nonunion site. However, two studies have reported disappointing results of bone healing following closed nonunion treatment with a success rate of 73% and 58%, respectively [15,16]. Functional deficits and physical impairment have been described in recent studies [17-19]. Thus, some authors concluded that the treatment of femoral shaft nonunion should include open resection of nonunion tissue and insertion of an auxiliary plate at the nonunion site to increase rotational stability [20,21]. The results of our study suggest that open nonunion treatment is not required and that closed treatment supports the concept of a biological osteosynthesis with preservation of soft tissue envelope and reducing the risk of secondary intraoperative infection or bacterial contamination. Predominately, this nonunion is a hypertrophic nonunion requiring additional stability. Insertion of increased nail diameter has several mechanical advantages. Penzkofer and coworkers demonstrated that with increase in nail diameter, the axial and rotational stability increased significantly [22]. This concept is supported by studies which found a positive correlation between reaming diameter and nail size larger at least in 2 mm than the primary nail with the fracture union rate [2,12]. Additionally, distribution of axial forces and compression at the nonunion site is very important. This can be ensured by applying dynamic compression with insertion of an interlocking and a compression screw. Postoperative unrestricted weight bearing also contributes to axial compression of the nonunion site.

A major concern of the reaming process is the detrimental effect of heat created by the drill bit resulting in necrosis and impairment of bone healing. However, beneficial effects of limited reaming have been described recently as reaming graft which has been shown to result in an improvement of local biology and stimulation of bone healing at the nonunion site. Additionally, the utilization of reaming debris instead of stem cells or autologous bone graft avoids extra surgery to harvest autologous material. Reaming debris contains viable osteoblast-like cells and growth factors and might thus act as a natural osteoinductive scaffold. In a sheep tibia model, bones treated with reaming debris demonstrated a larger callus volume, increased bone volume, decreased cartilage volume in the fracture gap, and increased torsional toughness compared to the empty gap group [23].

Previously, Shroeder et al. reported a series of closed, intramedullary exchange nailing with reamed insertion in a subgroup of 42 patients with femoral shaft nonunion after a previous intramedullary nail with an 86% union rate and with only 14% requiring additional procedures [13]. Similar results had been published earlier, demonstrating comparable high union rates after this procedure [8-12]. Less favorable results with success rates as low as 73% and 58%, respectively, have also been reported [15,16]. Reasons for this discrepancy may be the lack of systematic microbiological analysis. Even in the absence of macroscopic evidence of infection, a low-grade infection may contribute to treatment failure. We therefore routinely harvested multiple bacterial samples from the reaming graft as well as obtained intraoperative material for culturing in thioglycolate culture medium to rule out infections. Although the study was designed as a prospective study, the treatment concept of closed exchange reaming was applied to all patients and no alternative treatment concept was evaluated. Due to the lack of a control group, the results cannot be compared and our treatment concept cannot be evaluated by results of a control group.

## Conclusion

The concept of reamed exchange nailing and closed nonunion therapy resulted in reliable bone healing of aseptic femoral shaft nonunion and is considered the standard procedure of choice for this nonunion entity. A single operative procedure is sufficient to achieve bone healing almost all cases. Limited correction of axis deviation is possible even with closed nonunion treatment. The lack of open resection avoids additional surgical damage to soft tissues and follows the principle of a biological osteosynthesis with low operative morbidity. The increased stability resulted in high postoperative patient comfort and low pain sensation, allowed for early, unrestricted weight bearing, and active rehabilitation.
